# Conservation status and cultural values of sea turtles leading to (un)written parallel management systems in Fiji

**DOI:** 10.1007/s13280-022-01766-4

**Published:** 2022-08-06

**Authors:** Salanieta Kitolelei, Alisi Soderberg, Nemillie Qaqara, Shritika S. Prakash, Malakai Tuiono, Joeli Veitayaki, Susanna Piovano

**Affiliations:** School of Agriculture, Geography, Environment, Oceans and Natural Sciences (SAGEONS), The University of the South Pacific, Laucala Bay, Private Mail Bag, Suva, Fiji

**Keywords:** Customary management, Fishermen, Formal and informal institutions, Legislation, Sea turtles

## Abstract

Globally and locally, conservationists and scientists work to inform policy makers to help recovery of endangered sea turtle populations. In Fiji, in the South Pacific, sea turtles are protected by the national legislation because of their conservation status, and are also a customary iTaukei resource. Centered on our interview-based study at Qoma and Denimanu villages, parallel management systems coexist, where both the (written) national legislation and the (unwritten) customary iTaukei rules determine the time and the quantity of sea turtle harvest. In addition, non-governmental organizations and academic institutions may influence local sea turtle management by providing scientific awareness and helping divert the economic values from the meat to the living animal. We suggest that the government and non-governmental organizations emphasize community management of sea turtles, and work alongside the customary chiefs and their fishing clans to understand the real harvest (eventually by allowing quotas) and to monitor the recovery of South Pacific sea turtles in Fijian waters.

**Supplementary Information:**

The online version contains supplementary material available at 10.1007/s13280-022-01766-4.

## Introduction

Sea turtles are an imperative component of global biodiversity and numerous societies around the world (Arthur et al. 2008; Brikke 2009). Sea turtles cultural significance varies across the world, although a high cultural value may not deter a wide scale commercial trade (Allen 2007). In 1982, the International Union for Conservation Nature Red List^1^ classified all sea turtle species as *endangered*, except for the *vulnerable* loggerhead turtle (*Caretta caretta*) and the *data deficient* flatback turtle (*Natator depressus*). Fourteen years later, in 1996, the hawksbill turtle (*Eretmochelys imbricata*) and the Kemp's ridley sea turtle (*Lepidochelys olivacea*) were reclassified as *critically endangered* due to the overall decline driven by unceasing exploitation and other factors (Motimer and Donnelly 2008; Wibbels and Bevan 2019). Temporal investigation into the ecological significance and biology of sea turtles has informed an increased scale protection at the global level, with multilateral and regional agreements and national laws to protect sea turtle species across their range of distribution. Some of the notable global agreements include the Convention on the International Trade of Endangered Species of Wild Flora and Fauna (CITES), first signed in Washington in 1973, and the Convention on Migratory Species of Wild Animals (CMS), first signed in Bonn in 1979. In the South Pacific, the more recent regional guideline is from the Secretariat of the Pacific Regional Programme (SPREP) Regional Marine Species Program 2022–2026.^2^

### Status of sea turtle research in Fiji

Fiji waters are inhabited by four of the world’s seven sea turtle species; the green (*Chelonia mydas*), hawksbill, loggerhead and leatherback (*Dermochelys coriacea*) turtles (Piovano and Batibasaga 2020). Of these species, the green and the hawksbill turtles nest and forage in Fiji (Piovano et al. 2019; Prakash et al. 2020). The hawksbill turtle nests almost all-year round but has peak nesting in January, followed by a peak hatching in March (Prakash et al. 2020). Single paternity in Fijian hawksbill turtles was recently suggested based on genetic studies (Prakash et al. 2022). The origin of the green turtles foraging in Fiji ranges from Australia in the West to French Polynesia in the East and, based on the only two investigated foraging grounds of Yadua and Makogai Islands, the American Samoa management unit^3^ is the primary contributor (Piovano et al. 2019 and review therein). Seasonal recruitment is influenced by seawater temperatures and mostly occurs in summer (Piovano et al. 2020). The juvenile green turtles have well-differentiated morphological traits (Álvarez-Varas et al. 2021), are present in shallow coastal waters (Papale et al. 2020), and their mixed diet has a strong invertebrates component (Piovano et al. 2020).

Sea turtles are long living, slow maturing species, which have a high mortality rate as hatchlings and juveniles (Bjorndal 1985; Hoorn 2016). The ecological importance of sea turtles is linked to their feeding habits and highly migratory behavior ranking them as ecosystem engineers and keystone species (Allen 2007; Lovich et al. 2018). Feeding habits of sea turtles reduce jellyfish population and corallivores, reduce nitrogen in seagrass meadows, and increase the disintegration rates of mollusk and transferring minerals within and between the marine ecosystems (Bouchard and Bjorndal 2000; Lal et al. 2010). Moreover, on nesting beaches, sea turtles’ eggs introduce nutrients into beach ecosystems and contribute to stabilizing sand dunes critical for their reproductive success (Lovich et al. 2018). Nutrients that remain in the nest chamber after hatching contribute organic matter that feed bacteria, fungi, ants and crabs, among others (Dodd 1988). Nest contents can also be consumed by predators or absorbed by plants (Fowler 1979). Sea turtles further serve as dispersal agents across the vast migratory paths they travel between nesting and foraging grounds transporting nutrients, energy, and marine fauna and flora (Bouchard and Bjorndal 2000; Allen 2007). In addition, they may serve as a pathogen or parasite host as well as substrates for epibionts^4^ (Allen 2007).

### Cultural background and traditional knowledge of sea turtles in Fiji

For millennia, sea turtles (**vonu**) and the **iTaukei** (indigenous Fijian) communities interacted, resulting in sea turtles being embedded in the **iTaukei** culture as totems, spiritual deities, myths and chiefly tributes (Williams 1898; Morgan 2007). In pre-colonial times, sea turtle harvest was regulated by chiefs^5^ and the turtles capture was limited to a selected group of traditional fishermen from the traditional fishing clans (**gonedau**). These fishing clans had a strict division of labor among the fishers, canoe makers and net makers. Women did not participate in sea turtle hunting because customarily, it was taboo for them to be associated with turtle hunting. Additionally, in a traditional setting, **iTaukei** participate in a customary division of labor where men captured sea turtles for their chiefs while women would glean or target small fishes in nearby coastal areas. Specialized fishing nets called **lawasau** (Veitayaki 1994) were used. The custom of turtle net making was highly institutionalized by the **gonedau** families from a delegated clan, who made nets of coconut (*Cocos nucifera*) sinnet (**magimagi**) or *Hibiscus tiliaceus* (**vau**) and presented them to the paramount chief’s turtle fishermen.

Customary requests for sea turtle from the paramount chiefs to their turtle fishermen followed a traditional communication channel between several fishing clans, and when the turtle fishermen agree to the task, they organize a special kava ceremony for accepting the delegated task. The capture of sea turtles by fishermen would be reciprocated by chiefs with **tabua**^6^ (a whale’s tooth) and food offerings of yams, taro and pigs (Tippett 1968; Erasaari 2013). Respect for taboos and beliefs associated with it, as well as the restricted access options in pre-modern times limited unauthorized consumption of this chiefly resource, locally protecting sea turtles from overexploitation for centuries. Some beliefs include children being born with disabilities if their mothers consumed turtle meat during pregnancy, or clans getting sick or dying if they consume turtle meat without permission (A. Mataitini, *pers. comm.* to SK; M.M. Lomaloma, *pers. comm*. to JV). In those times, elaborate rituals were employed before and after sea turtle captures (Toganivalu and Hunter 1913). Consumption of sea turtles was restricted to chiefs (and sometimes warriors) although, later, the chiefs used sea turtles as tributes to the missionaries and, in the 1830s, Christian **iTaukei** were allowed to consume sea turtles during a church construction or the opening of a church (Williams 1898). Customarily, sea turtles were used as head of tribute^7^ during ceremonies such as a chief’s birth, wedding or death (Deane 1910; Thompson 1938).

Sea turtles are totem fish^8^ for the people of Nacamaki in Koro and Kadavu, some clans in Taveuni, Galoa in Bua and some clans in Serua, Tuvuca in Lau, Malolo and Malake (M. Tuiono and T. Tikoibua, *pers. comm.* to SK) (Map S1), and their consumption within these communities is strictly forbidden. Sea turtles in Fiji are still exclusively harvested only by men (Veitayaki 1994; Kitolelei et al. unpublished data). Traditional knowledge of **iTaukei** fishermen includes the nesting and fishing seasons of sea turtles, both of which are incorporated into traditional fishing calendars (Kitolelei et al. 2021). For example, **iTaukei** fishermen know that sea turtles nest during the **Vula i Katakata**^9^ (summer months, from November to March) (J. Bogidrau, *pers. comm*. to SK). Traditional taxonomic classification demonstrates the cultural prominence of sea turtles in Fiji as the *ethno-species*^10^ are named by fishermen according to turtle’s life stage, color, and sex (Table 1). The great ecological and cultural significance of sea turtles is also reflected in Fijian school children’s drawing of the sea, where they are prominently present (Fache et al. 2022).

**Table 1:** Ethno-species of sea turtles in Fiji as defined by iTaukei fishers from Fiji contributing to the traditional classification for sea turtles. *Sources*: field data collected by the USP Turtle Team from Yadua (2015), Kitolelei S. from Verata Ucunivanua, Yadua and Qoma (2020), interview with Paul Geraghty (13 April 2021), and references to an unpublished iTaukei-iTaukei dictionary *Ivola vosa* by Geraghty 2020. (Juv. = juvenile)

Scientific name	Common name	Ethno-species
*Chelonia mydas*	Green sea turtle	**Vonu dina, ikabula dina, ikadina, ikadu, maloi, dakarosawa, balakaisovu, todoro, vonu damu** (juv.)**, ikadamu** (juv.), **guru**, **mokoloa** (adult), **bala** (male), **mino** (female that nest), **balakaisovu (**juv. male**)**, **todro, bicinidevo** (juv.), **maladamu** (juv.)**, tabadamu** (juv.)**, vonumatanisiga** (juv.), **tavatavadraunitiri** (newly hatched), **tavatavadraunidogo** (newly hatched)
*Eretmochelys imbricata*	Hawksbill turtle	**Taku, vonu taku, taku loa, taku damu, taku vula, batitolau, batibati niuniu, noco, takona, vonu ta’u**
*Caretta caretta*	Loggerhead sea turtle	**Vonu, ikabula, balabala, tuvonu, seleniwai, serevahi, vonu ni Toga, vonu ni Lau**
*Dermochelys coriacea*	Leatherback turtle	**Vonu, ikabula, vukitabaiwalu, tanoa, tabaiwalu, tewenivonu**

The sacredness of sea turtles started to diminish in the 1840s with the beginning of the tortoiseshell trade in Fiji, when sea turtles became part of the cash economy (Williams 1898; Veitayaki 1994). The **iTaukei** were encouraged to preserve turtle shells, and when the sandalwood and sea cucumber industries failed in 1865, the tortoiseshell trade began with US traders (Williams 1898). The custom of capturing sea turtles only upon a chief’s request fell into disuse, substituted by a widespread exploitation to cater for the tortoiseshell demand and the subsistence or commercial use of meat and eggs (Tippett 1968; Guinea 1993). Colonial influence further changed **iTaukei** customs (Toganivalu and Hunter 1913; Muehlig-Hofmann 2008), and custom-based norms and rules which limited the exploitation of sea turtles died out and, for about a century, a widespread unhindered sea turtle harvest in Fiji occurred (Guinea 1993). To limit the exploitation and its consequences, the (not yet independent) Fijian Government introduced a regulation of sea turtle harvest in the Fisheries Act in 1941. More recently, a legal prohibition of sea turtle harvest was put in place in 1995, followed by three moratoria,^11^ which collectively prohibited sea turtle molesting, taking or killing as well as harvest of sea turtle eggs from 1997 to 2018. These moratoria acknowledged the customary rights of the **iTaukei** and reserved them the option to apply for a special “exemption for traditional use” permit from the Ministry of Fisheries, although an application does not necessarily grant a permit. Today, sea turtle protection in Fiji falls under Regulation 5 of the Offshore Fisheries Management Regulation 2014 (OFMR) and the Endangered and Protected Species Act 2002 (a legislation that implements CITES in Fiji). The Fisheries Act and other laws governing sea turtle harvest and use do not integrate the unwritten **iTaukei** customs which relate to sea turtles.

This study investigates how two Fijian communities of traditional turtle hunters cope with parallel systems (written law policies and unwritten customary rules), and explores the integration of the traditional knowledge with conservation and management of the sea turtles.

## Materials and methods

### Study sites

**Fig. 1:**
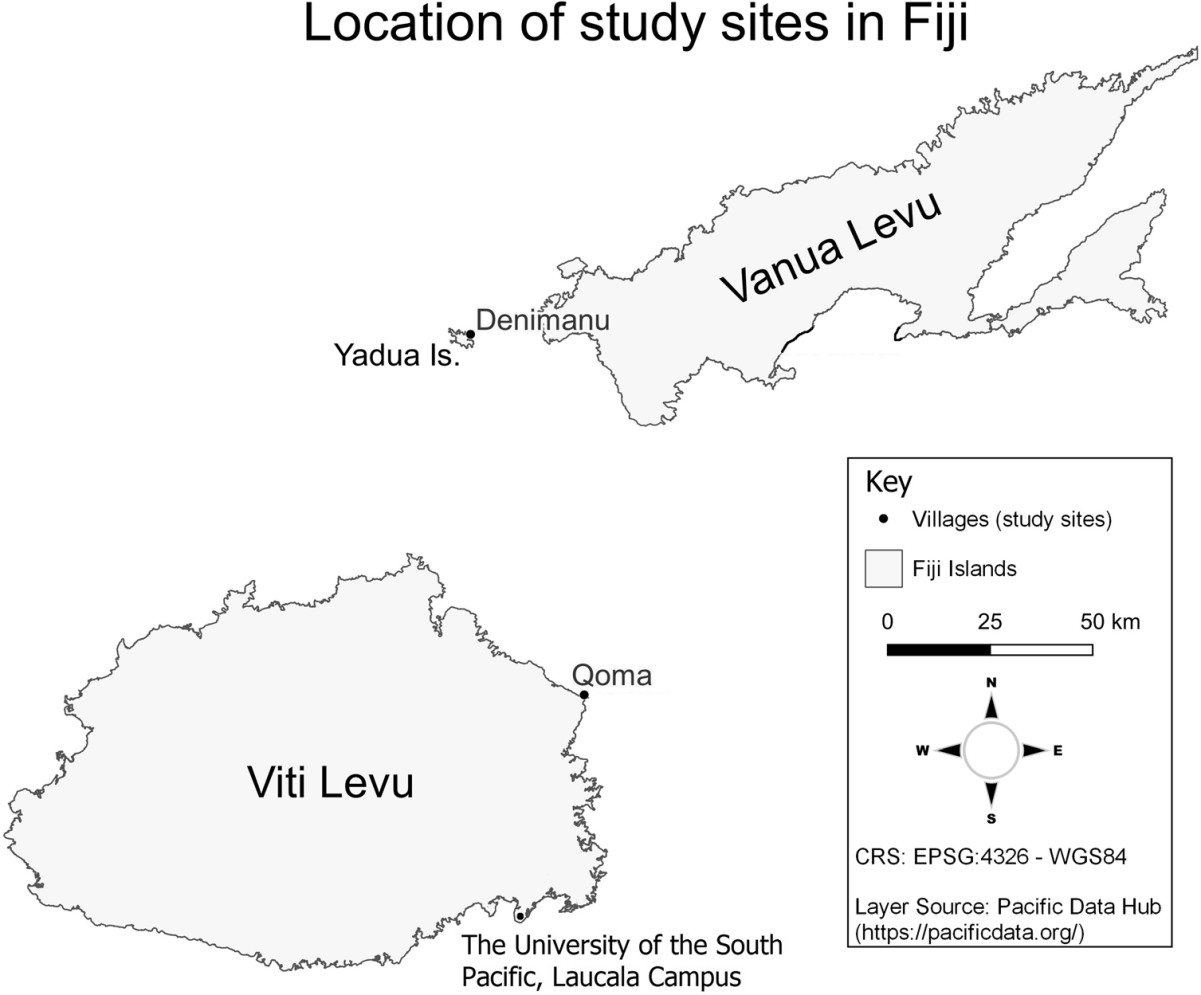
Map of the three Fijian study sites: Qoma village (Nabulebulewa and Qoma Levu Islands), Denimanu village (Yadua Island) and The University of the South Pacific (USP) Laucala Campus (Suva)

Geographically, the study sites (Fig. 1) include USP Laucala Campus in Fiji’s capital city of Suva, and two rural coastal villages with traditional fishermen: Qoma village, where the unwritten customary rules are cited to still catch sea turtles (Kitolelei et al. unpublished data), and Denimanu village, where protection of sea turtles has led to strict adherence to the written national legislation on sea turtle conservation and to an adaptation (at least, temporarily) of the unwritten customary rules.

Qoma village is located on the northeastern coast of Viti Levu and its population is spread between Qoma Levu and Nabulebulewa islands. Qoma shares its customary fishing area (**iqoliqoli**) with neighboring villages of the same district (**tikina**). No sea turtle nesting activity is reported from this site (Prakash et al. 2020). The area is known for its green, hawksbill and loggerhead sea turtles (Veitayaki 1994). Its **gonedau** fish for the Paramount Chief (**Turaga na Ratu**) of Ucunivanua in Verata.

Denimanu village is on Yadua Island, a hawksbill turtle nesting site (Prakash et al. 2020) located west of Vanua Levu. Its **iqoliqoli** hosts green and hawksbill turtles foraging grounds (Piovano et al. 2019; Papale et al. 2020), as well as loggerhead sea turtles (P. Qarau *pers. comm*. to MT). Denimanu fishermen, the **gonedau** of Bua, are customarily obliged to catch sea turtles for the chief of Bua (**Tui Bua**). However, they are also part of the **Dau ni Vonu** (DnV, ‘guardians of the turtles’) network, which was set up in 2010 by the World Wildlife Fund for Nature (WWF) with the aim of increasing the number of sea turtles before resuming hunting. Denimanu village’s commitment to manage and conserve sea turtles is still on-going. Whenever possible, the villagers actively participate in the USP’s sea turtle research project, as well as in non-governmental organizations (NGOs) conservation projects.

### Data collection and analysis

Data for this study was collected by combining six methods (Table 2). Briefly, (1) In-depth interviews with elder fishermen were conducted in Qoma and Denimanu in the summer of 2020. Elders were chosen because the interviews focused on gaining insights on the temporal changes in sea turtle fishing since the 1950s. (2) Open-ended questionnaires, which were given to Denimanu sea turtle fishermen in February and November 2015, with the aim of reviewing sea turtle fishing habits in over a 30-year period. (3) Informal conversation (**talanoa**) sessions with **iTaukei** staff and students (both men and women) at the University of the South Pacific (USP) who were willing to participate after the research was explained to them. **Talanoa** were conducted to gauge the use and value of sea turtles during traditional ceremonies. Participants were chosen according to their availability at the time of the **talanoa** (4) A two-question online survey, done via SurveyMonkey^12^ to ask the public about the last time they witnessed a sea turtle as the head of tribute, and if that ceremony was associated with a hereditary chief. A link to the survey was shared via social media (Facebook and Twitter). The survey was opened from 19 to 26 October 2021. The questions were asked to gauge how the sea turtle value as head of tribute in chiefly ceremonies changed. (5) Semi-structured interviews, which were conducted in Qoma in 2017 and 2018 with the aim of gaining any change in the perceptions of sea turtle hunting. (6) In-depth questionnaire to focal groups was administered to two male groups, two female groups and one youth group to collect traditional knowledge and fishing background of sea turtle harvest in Qoma and Denimanu. Although women were not involved in sea turtle hunting, they were included in the survey because they actively took part in food preparation, particularly in the cooking of sea turtle meat for feasts.

**Table 2:** Data collection methods used in this survey. Target population key: FM = fishermen; FW = fisherwomen; TF = turtle fishers only; E = elders (60 + years old); P = public; iT = iTaukei Marine staff and students

Location	Method	Date	Total no. interviewed	Target population	Male	Female	Survey Team
Qoma	Semi-structured interviews	(1) Nov. 2017 (2) Nov. 2018	18	FM, FW	11	7	NQ
Qoma	In-depth interviews and focal groups	Nov. 2020	33	E, FM, FW	18 (60 + years old = 3)	15 (60 + years old = 3)	SK, AS
Denimanu	Collected questionnaires	(1) Feb. 2015 (2) Nov. 2015	37	TF	37	0	SSP, SP
Denimanu	Questionnaire-based interviews & focal groups	Sept. 2020	38	FM, FW	15	23	SK, AS, SSP, MT
USP	**Talanoa** (informal storytelling)	Apr. 2021	43	iT	22	21	SK, MT
Online survey	Survey Monkey two-question survey	Oct. 2021	60	P	N/A	N/A	SK
Total			229		103	**66**	

A qualitative analysis was carried out on the answers given by the 229 participants (51 in Qoma and 75 in Denimanu; Table 2) whose age ranged from 18 to 85-year-old.

## Results

A summary of the results is given in Table 3 and expanded in this section. The results are presented by subject and aim to compare the two villages of Qoma and Denimanu.

**Table 3:** Summary of the similarities and differences in the Results presented in this manuscript

Results	Similarities	Differences
Target species	BOTH Qoma and Denimanu fishers target green and hawksbill turtles. Green sea turtles are more preferred for their tasty meat.	Qoma fishers left eggs alone because they were considered sacred. Denimanu fishers collected hawksbill turtle eggs for subsistence use until 2000.
Fishing methods	BOTH Qoma and Denimanu fishers used net fishing and spear-fishing to capture juvenile and adult sea turtles.	Qoma fishers perform a turtle hunting ritual and adhere to a strict set of rules for the duration of the turtle hunt. Denimanu fishers captured sea turtles (whenever they were spotted prior to the turtle legislation)
Chief’s decision-making	The chief makes the decision in turtle harvest which occurs in BOTH Qoma and Denimanu.	The Qoma chief’s decision is based on the needs of the community. The Denimanu chief’s decision is based on the importance of the ceremony, abundance of sea turtles and availability of substitutes such as pigs or cows.
Role as traditional turtle hunters for paramount chiefs	BOTH Qoma and Denimanu turtle hunters are **gonedau** (traditional fishers) for paramount chiefs in their respective Districts. Therefore, they are traditionally obliged to present sea turtles as the head of their tributes. The paramount chiefs of these two areas adhere to the sea turtle legislations, therefore discourage the harvest of sea turtles.	
Sea turtle capturing season	BOTH Qoma and Denimanu fishers target sea turtles during their mating and nesting periods.	Qoma fishers still capture sea turtles but only after they get permission from their chief. Denimanu fishers capture and release sea turtles for monitoring and conservation purposes during their nesting season.
Response to legislations	BOTH Qoma and Denimanu fishers are aware of the sea turtle legislations	Qoma fishers continue to capture sea turtles with or without licenses. Denimanu fishers capture sea turtles only after they get both license and approval from their chief

### Target species and fishing methods

Hawksbill and green turtles were targeted by the respondents in both Qoma and Denimanu. The fishermen of Qoma primarily targeted green turtles. In the past, sea turtle eggs were left untouched because nests were located on inaccessible shores and sea turtles were considered sacred. In order to harvest sea turtles, fishermen in Qoma had to seek permission from their chief and sub-clan (**tokatoka**) by formally presenting **yaqona**,^13^ and had to follow hunting taboos such as: (i) fishermen must not make unnecessary noise during the duration of the sea turtle fishing, (ii) abstaining from having intimate relations with their wives or women, from the time the sea turtle hunting is decided to the end of the sea turtle hunt, (iii) stealing and infidelity forbidden, (iv) when out at sea, turtle hunters are not allowed to eat products from land until they return from their trip, and (v) any money or gifts received in exchange for the sea turtle harvested is shared equally amongst members of the household including visitors present at the time of the exchange. To fulfil customary and religious obligations, each of the seven **tokatoka** in Qoma has to contribute one sea turtle at Christmas, Easter and wedding celebrations, and three per **tokatoka** for an annual church feast. This fulfillment would be done irrespective of obtaining a permit from the Ministry of Fisheries.

In Denimanu, prior to the year 2000, the fishermen altogether would annually collect an estimated 700 eggs of hawksbill turtle, for subsistence only. With an average of 121 eggs per nest (Prakash et al. 2020), this roughly equates with six nests. Fishermen harvested whole clutches. During the nesting season, customary bans were placed on the harvest of nesting females, therefore, eggs were targeted. The practice of egg collection disappeared entirely by the year 2015 (Table 4), and today nests are located and estimated hatched eggs are counted by the DnV in collaboration with NGOs that contribute data for sea turtle population assessments. Green turtles were targeted prior to 2000 for their meat and were sought after both as chiefly tributes (**magiti ni kakana vakaturaga**) and for subsistence use, because the villagers “preferred the taste of green turtle’s meat over the hawksbill turtle’s and because "it had more meat than fish”. According to the respondents, the **Turaga ni Vanua** decision to protect sea turtles to increase their numbers in the village waters has led to the village participation at the **Dau ni Vonu** network and to the village strict adherence to the sea turtle national legislation. As a result of this effort, an 80% reduction in green turtle captures was achieved. From 2000 to 2009 sea turtles were only captured as tributes for a few traditional chiefly ceremonies and weddings. In 2009, sea turtle harvest was declared taboo by the **Turaga ni Yavusa**. Requests for exceptions could be presented to the chief and, upon the chief’s approval, the customarily authorized fishermen must obtain a permit from the Ministry of Fisheries before the harvest. However, whenever possible, customary tributes of sea turtles are now substituted by the presentation of a live pig or cow as chiefs adhere to the turtle legislation and capturing sea turtles is now harder due to overfishing.

**Table 4:** Summary of sea turtle capture activities, legislation adherence, permit requests and customary permissions granted by chiefs for sea turtle harvests in Qoma and Yadua over the past 40 years. Source: data collected from field research by authors of this paper (2015–2021). *Fishing methods: NF = net fishing, HC = hand collection, SF = spear fishing. The number of fishing techniques named are included in a bracket next to the fishing method

Village	Species	Life stage	Fishing method (No. of fishing techniques)	Uses by villagers	Last reported harvest (Qty.)	Awareness of legislations	Permit required	Chief’s permission required
Qoma	Hawksbill	Eggs	Hawksbill eggs left untouched					
		Juvenile	NF (6)	Food, shell curio, fundraising	2020 (1)	Yes	No	Yes
		Adult	NF (6), SF (2)	Food, head of tribute for chiefly ceremony, wedding, funeral, birthday, subsistence, fundraising	2020 (2)	Yes	Yes	Yes
	Green	Eggs	Green eggs left untouched					
		Juvenile	NF (1), SF (1)	Subsistence	2020 (1)	Yes	No	Yes
		Adult	NF (1)	Head of tribute, chiefly ceremony, wedding, fundraising	2020 (3)	Yes	Yes	Yes
Denimanu	Hawksbill	Eggs	HC (1)	Subsistence (targeted during nesting season when turtles harvest is taboo)	2000 (714)	Yes	No	Yes
		Juvenile	HC (2)	Subsistence	2000 (1)	Yes	Yes	Yes
		Adult	HC (1)	Head of tribute, subsistence	2000 (13)	Yes	Yes	Yes
	Green	Eggs	N/A	No green turtle nesting site on Yadua Island	N/A	N/A	N/A	N/A
		Juvenile	HC (2), SF (1)	Subsistence use	2000 (5)	Yes	No	Yes
		Adult	HC, SF (1)	Head of tribute, subsistence	2000 (27)	Yes	Yes	Yes

Sea turtle harvest involves two fishing methods and several techniques. In Qoma, fishermen use six net fishing techniques and two spear fishing techniques. In Denimanu, fishermen use one spear fishing technique, one net fishing technique and two specialized target species methods (Table 4). The turtle fishing methods used by fishermen were influenced by the tide, diurnal cycle, fishing location and the purpose of fishing (Table S1).

### Temporal changes to customary values of sea turtles

According to an elder in Ucunivanua, an ancient communication channel existed between the paramount chief in Ucunivanua, **Mataqali** (clan) Saraviti in Naloto village, and the Qoma sea turtle fishermen. This channel is no longer used as the paramount chief in Ucunivanua complies with the sea turtle legislation. Older fishermen who were interviewed in Qoma and Ucunivanua detailed the sacredness of sea turtles as they recollected stories from their grandparents and described how sea turtles were heads of tributes for a hereditary chief’s funeral or wedding and captured only upon request of the paramount chief of Ucunivanua. Such sea turtle capture by fishermen in Qoma stopped in the 1950s, and today a majority of the sea turtles harvested in Qoma are no longer restricted to events for hereditary chiefs. Sea turtle presentation and consumption are for weddings, fundraising events or house construction involving the whole community. Strict customary norms and rules and recent low catch rates (Table S2) made sea turtle fishing nowadays less attractive to Qoma’s fishermen who, nevertheless, are now fishing for sea turtles for both economic and subsistence uses.

In Denimanu, sea turtles were formerly hunted for major communal events, such as weddings or a chief’s funeral, and presented as tribute for the **Tui** Bua. Prior to the 1960s, turtle hunters captured sea turtles during their nesting period. When a fisherman spotted a sea turtle while out on a fishing trip, that fisherman returned to the village, informed the other fishermen and a turtle hunt was organized by selected fishermen from the **gonedau** clan. Exploitation of sea turtles in Denimanu spiked, leading to a reduction in sea turtle population between the late 1960s and 1995, and this decrease is attributed to the introduction of fiberglass boats, nest destruction when collecting eggs, climate change and the deterioration of customary taboos on sea turtle captures. In 1995, due to the annual national ban on sea turtle capture, Denimanu villagers slowly began to wean themselves off the consumption of sea turtle meat. A ban imposed by the chief in Denimanu and a community-based initiative to protect the sea turtles made response to the sea turtle conservation efforts easier for the fishermen in this site.

**Fig. 2:**
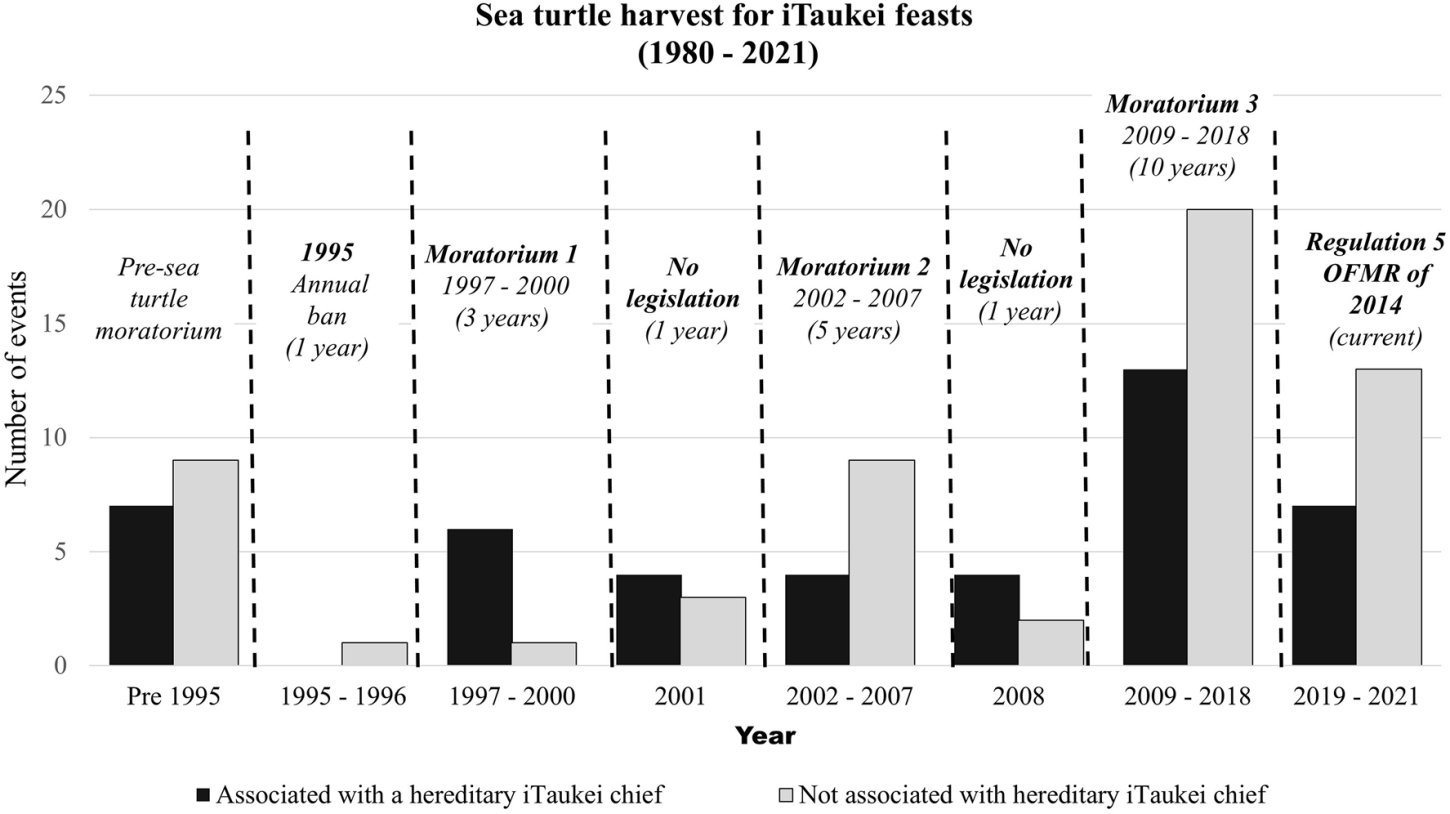
This graph shows responses from random interviewees on their last memory of when sea turtles were presented as the head of tribute for an iTaukei feast. The answers were separated as associated with a hereditary chief and not associated with a hereditary chief. Source: Unstructured interviews at USP and online survey via Survey Monkey Audience

Information collected through **talanoa** sessions and online survey span over 41 years (1980–2021) show that after the 1995 annual ban on sea turtle harvest and in between moratoria (2001 and 2008), sea turtle harvest was dominated by activities related to a hereditary chief. However, during periods where the moratoria were active, the majority of the sea turtles harvested were not associated with hereditary chiefs (Fig. 2).

### Response to legislations, customary law, and third-party interference

Interviewees in Qoma and Denimanu were all aware of the three moratoria protecting sea turtles (Table S2). In Qoma, sea turtle harvest only occurred when approval is given from the chief and sub-clan, with or without sea turtle harvest permits. However, the harvest is approved only if the purpose is stated and their sub-clans agree on sharing the benefits equally in the village. The leaders of the sub-clans ensure that the sea turtles are harvested and used for the purpose initially stated when permission was granted. It has been noted that the requirement of seeking permission in the national sea turtle legislations is substituted by the customary rules which community members adhere to and are monitored within communities.

In Qoma, legal and illegal sea turtle fishing occur together, with permission from their community chief. This choice is based on their belief that enough sea turtles will present themselves for capture only if the fisherman urgently needs them and that transparency is used when sharing the monetary benefits of the catch. Furthermore, turtle sales have become lucrative for Qoma fishermen and their primary income source or subsistence item, and this at times has caused conflicts between the fisherman and community members who adhere to the legislation.

In Denimanu, 40% of the fishermen agreed that the 26 years of sea turtle protection (1995–2021) resulted in an increase in the number of nests, of nesting females and of juvenile sea turtles in their fishing grounds. While sea turtle harvest was monitored by the DnV and the chief in Denimanu, anyone who needed to harvest a sea turtle required a permit from the Ministry of Fisheries prior to the chief’s consent. During the annual ban on sea turtle harvest in 1995, there were no sea turtle captures, and during the first Moratorium, sea turtle harvest was limited to chiefly functions. Most of the few sea turtles harvested in Denimanu since 2000 have been used communally and not for individual subsistence consumption like it was prior to 2000.

Despite the contrasting behavior between these two villages, the last eighty years (1941–2021) have witnessed major reductions in sea turtle harvest in both Qoma and Denimanu (Table 5) when compared to anecdotes from older fishermen. Apart from the government and the community, third parties which are not linked to communities or the government such as non-governmental organizations (NGOs) and academic institutions have played a significant role. In Qoma, interviewees have learnt about the ecological importance of sea turtles from NGOs/academic institutions and, in turn, the villagers have educated them on the cultural value of sea turtles to the people of Qoma. However, the new knowledge did not change the fishermen’s behavior.

**Table 5:** Combined effectiveness of sea turtle legislation, moratorium, strict customary protocols on turtle harvest and the Dau ni Vonu network at Denimanu in Yadua Island, Fiji, South Pacific, shows a clear reduction in the numbers of sea turtles captured in the year 2000 compared to the year 2015. Estimates are from information in the Denimanu questionnaires of 2015

Ceremony or feast	Hawksbill turtle (*Eretmochelys imbricata*) vonu taku			Green turtle (*Chelonia mydas*) vonu dina		
	2000	2015	Total_1_	2000	2015	Total_1_
Weddings	85	35	120	122	27	149
Birthdays	53	5	58	99	5	104
New Year Celebration	58	0	58	110	12	122
Christmas	53	0	53	110	5	115
Easter	45	0	45	108	0	108
Funerals	79	10	89	115	0	115
Grave cementing	66	0	66	95	0	95
Ivakananumi (Church memorial day)	55	0	55	98	0	98
Chief’s request	7	10	17	40	0	40
Others: Sevu*, gravesite cleaning (cara bulubulu)	1	0	1	41	10	51
Total_2_	502	60	562^+^	938	59	997^+^
84% total reduction in the number of sea turtles harvested between 2000 and 2015	79% reduction in the harvest of hawksbills between 2000 and 2015			88% reduction in the harvest of greens between 2000 and 2015		

*Sevu refers to the annual offering of harvests presented to the Church which is usually done in February (**Vula I Sevu**) on the traditional **iTaukei** calendar. Gravesite cleaning is done annually before Christmas. Total_1_ refers to the total number of turtles (two species) caught per ceremony/feast. Total_2_ refers to the total number of turtles caught per year (2000 and 2015). *N*^+^  = total number of turtles harvested by species as reported in the questionnaires. Note: these estimates are based on interviewees’ memories, as there is no written record of the harvest

In Denimanu, SPREP and WWF’s sea turtle conservation initiative successfully converted sea turtle hunters to conservators, based on the idea to reach a population that can be sustainably harvested. Their presence in Denimanu has influenced a 79% reduction of hawksbill turtle harvest and 88% reduction in green turtle harvest between the year 2000 and 2015. Following this, extensive work has been undertaken by USP. Denimanu as a community incorporated an “adaptive management” strategy in their sea turtle management, based on the combination of local knowledge and skills as turtle hunters with the scientific information provided to them to manage the foraging and nesting areas of sea turtles around Yadua Island.

Finally, in both Qoma and Denimanu, interviewees reported that Christianity influenced their decision to harvest sea turtle. This means that their faith influenced some beliefs associated with sea turtle consumption such as punishment for secretly eating sea turtle meat without a chief’s permission.

Generally, the responses to all the data collection methods were similar, as they all mentioned the importance of sea turtles to the **iTaukei** culture. They also mentioned the scarcity of sea turtles and how the sea turtle legislations are known to them.

## Discussion

There is a growing literature exploring the conflicting situations that can arise when (written) law or policies influenced by Western knowledge are in contrast with (unwritten) traditional indigenous customary rules. Excessive harvesting of the living resources has led to a decline of several species by the mid-twentieth century, after which ‘a period of prohibition, control, and regulation of wildlife use that produced positive results in a few cases and places but failed terribly in the majority followed’ (Larriera 2022, p. 2). Worldwide, legal prohibition and penalties fixed by written policies were effective only where there was a high enforcement capacity, otherwise illegal harvesting would occur (Ingram et al. 2022). In the case of sea turtles, commitments to conservation started by adhering to international policies (e.g., CITES), upon recognizing a local population decline. Among the successful instances are systems allowing for monitored but legal commercial harvest; for example, a community cooperative in Costa Rica run a managed olive ridley egg harvest program for over 35 years (Campbell et al. 2007). Some countries adopted a different approach; for example, Aboriginal and Torres Strait Islander communities are legally entitled to harvest sea turtles for personal, domestic, and non-commercial use.

In recent times, a “compassionate philosophy” against animal use has developed in the urban centers of the Western countries (Griffin et al. 2020). On the contrary, Indigenous people in tropical Pacific and worldwide are increasingly claiming their traditional rights to harvest local resources (Beltran and Phillips 2000). Community-based management involving both people with and without decisional power, and integrating scientific knowledge (particularly of population dynamics) with traditional knowledge, proved to be effective in resource management and restoration (see for example the Locally Managed Marine Area Network^14^). Conservation through sustainable use, tailored to local situations and implemented via adaptive management, can be the way forward to ensure a sustainable use of the marine resources that is also respectful of local culture and traditions (Webb 2002). For example, in the case of crocodilians, there are several instances across the Southern Hemisphere where modern management of crocodiles was successfully carried out by integrating local traditional values (Brackhane et al. 2019). However, sea turtles are listed in Appendix S1 of CITES, consequently the signatory Countries are not allowed the commercial use of this traditional resource. In such a case, we suggest considering the inclusion of traditional and/or subsistence use. For example, based on our findings of the turtles’ customary importance in Fiji, we suggest a quota system of ten sea turtles (**dua na bi**^15^) annually for each of the seven paramount chiefs.^16^ Based on the current (although illegal and unreported) national annual harvest, we consider that reducing the captures to seventy juvenile specimens per year is a step towards the conservation goals. If the sea turtles that are captured are not used, they can be kept in tidal pools or traditional tidal pens and carried over to the following year. This quota system can be monitored through a collaboration between fishermen, fish wardens, fisheries officers and NGOs. This system could work in Fiji because it accounts for traditional obligations of fishermen to their paramount chiefs and for communal feasts. It also gives a chance for slow integration of conservation and monitoring efforts into the rural communities, and for recruiting more DnV members across Fiji’s customary fishing communities.

Customary law which regulates the use of resources at the local level is governed by customary authority and is vital in sustaining resources (Veitayaki 2000; Clarke and Jupiter 2010). Successful customary management practices depend on the strong leadership, monitoring and enforcement of regulations and social networks (Gutiérrez et al. 2011). When legitimate leaders inspire changes which are guided by the collective interests of the community, their decision-making enhances the community’s compliance to regulation changes and a community’s resilience as they go through governance changes (Olsson et al. 2004). When a community acknowledges a leader, they impact the strength of their leader’s influence and the obedience of the community (Sutton and Rudd 2016); which impacts on the success of conservation outcomes (Ison et al. 2021). For example, in Denimanu village, turtle fishermen comply to the turtle legislation because of the ban placed on sea turtle harvest by the chief and the community-based initiative to protect sea turtles. Community-based management initiatives which seek collaboration and adaptive management can work where communities are actively involved in resource management which are based on their communal management goals. Examples from the Pacific Islands include the collaborative and bottom-up structure set up in Palau for sea turtle management (Risien and Tilt 2008), community-based management of leatherback turtles in Solomon Islands (Jino et al. 2018) and the community-based management of nearshore fishers in Vanuatu (Léopold et al. 2013).

Reports of failed community-based management are often linked to their lack of association with higher level governance and related enforcement, resulting in outsiders entering a managed territory and exploiting the resources therein; such as the case of the Gulf of California, Mexico, where a locally created and enforced network of marine protected areas led to an increase in fishing resources’ abundance which, in turn, attracted poachers who were not bound by the local harvesting rules, resulting in overexploiting and rapid declines of the resources (Cudney-Bueno and Basurto 2009). Furthermore, marine resource use, management and conservation issues are complex (Ison et al. 2021) because of their multi-dimensioned nature, where the community, government, NGOs and academic institutions collaboration play key roles in strengthening conservation.

Organizations outside of the community and government often enable marine conservation by creating opportunities and tools which motivate conservation such as advocating concern for marine issues, providing scientific expertise, implementing conservation plans, enforcing management strategies and enabling the facilitation and capacity building in communities (Sutton and Rudd 2016). A collaborative environment which brings together public policy decision-making and positively engages people in public and private agencies, government and the civic society, creates an attitude of ethical engagement, communal motivation and the ability for collective achievements (Emerson et al. 2012). This is shown in Canada where two environmental NGOs work alongside government and communities by transmitting scientific data and information from researchers to decision makers therefore, advancing policy and practice (Cadman et al. 2020).

Conservation efforts or compliance to legislations fails in communities if the community and the leader do not support the initiative (Adams and MacShane 1996). Furthermore, Indigenous societies believe that culture defines nature, its value, as well as its conservation (Campbell 2002). As shown in this study, Qoma fishermen and their chief place the cultural value of sea turtles ahead of conservation efforts and legislation compliance. In addition, some Qoma fishermen believe the sea turtles are a God-given limitless resource (Kitolelei et al. unpublished data). An intersection between culture and conservation provides complexities in effectively regulating the conservation of sea turtles. A similar case is shown in the Caribbean Coast of Nicaragua, where conservation efforts by Wildlife Conservation Society aligned to the global sea turtle conservation is rendered ineffective because local communities traditionally harvest and consume green sea turtles (Conte 2011). In Denimanu, giving up the traditional right to harvest sea turtles is a choice the fishermen and their chief are willing to temporarily accept to ensure that their future generations will enjoy sea turtle sightings and harvesting. Realization that sea turtles are a resource with limits encourages Denimanu fishermen to protect sea turtles from overexploitation and to act to help the populations’ recovery. The genuine request to protect sea turtles from local communities ensure that the legislation adherence is enforced and sea turtle populations are monitored, as it is occurring in Denimanu.

Integrating unwritten rules into formal governance of marine resources contributes to management which is better equipped for future change (Pellowe and Leslie 2020) and finds common ground between everyone working in conservation. The essential foundation for collaborative governance of marine resource conservation is creating trust (Rapp 2020) which is built on values and integrity of individuals in achieving the same goal. For example, based on our findings, multiple efforts carried out by NGOs and USP can lead to observance of the national sea turtle policy by the local fishermen and the creation of a trust relationship between the sea turtle biologists and managers. Furthermore, for Denimanu village, it has led to continued efforts in protecting sea turtle nursery and feeding grounds at Yadua Island. Denimanu turtle hunters, who are in constant contact with sea turtles, are the local experts who possess key information on the health and status of sea turtle feeding habitats, nesting sites and habits. Acknowledging that their sea turtle populations have decreased over the years demonstrates a deep understanding, which led them to act to ensure that their future generations are able to have the same opportunities to catch sea turtles at their foraging grounds and to view sea turtles nesting on their island shores. We suggest the documentation of traditional knowledge and customary rules linked to sea turtle conservation, and their integration with scientific findings and research so that decision makers will make better informed recommendations.

However, in Qoma fishermen ignored marine conservation awareness efforts brought to their community by Government and NGOs and, instead, provided their own insights into the value of sea turtles to them as a **gonedau** clan for the paramount chief in Ucunivanua. In a customary setting, obligations are shared between the inland-based clans and those along the coast, where the presentation of tributes to a paramount chief obliges the inland-based clans to provide land-based resources and the coast-based clans to provide marine resources. The legally mandatory absence of sea turtles as a head of their tribute forces the **gonedau** clans to choose an alternative meat tribute, such as pigs or cattle, removing the customary live marine resource presentation delegated to the fisher clans. Accepting the legislation would make redundant their role as **gonedau**, and would be disrespectful to their paramount chief. Qoma turtle fishing is self-regulated because fishermen believe a sea turtle would allow itself to be captured when the fishermen need them – irrespective of it being for customary or economic purpose. We recommend a review of the existing policies to account for the traditional cultural role that iTaukei place on sea turtles as a way to convince ‘reticent’ villages, and thus to improve sea turtles management and conservation in the country.

Finally, our findings show the influential roles paramount chiefs and village chiefs still have in mobilizing the fishermen to monitor the sea turtles and spread awareness on the proper management of sea turtles in rural areas. A respected traditional leadership is a key factor in the success of locally managing marine and aquatic resources (Muehlig-Hofmann 2008). In both Qoma and Denimanu, the approval of the currently installed chief to harvest sea turtles carries more weight for turtle hunters compared to the permit given by the Ministry of Fisheries. Moreover, whenever a paramount chief requires sea turtles as a tribute, their **gonedau** will be informed through a traditional messenger, and they will carry out their duties as turtle hunters and present their catch to the paramount chief. This relationship between the **gonedau** and the chiefs is not dictatorial but the **iTaukei** custom of **veivakamenemenei** (act of showing loyalty to the chief), which is still practiced in many **iTaukei** communities today. We suggest that paramount chiefs, their **gonedau** and the village chiefs be offered the opportunity to witness the results of effective sea turtle protection programs, like the one on Yadua Island in Bua.

## Conclusion

Formal policies which govern marine resource use are important in shaping conservation. The correct translation and interpretation of these policies to resource users is vital for the compliance, particularly when dealing with culturally important resources such as sea turtles. Collaborative efforts between all stakeholders needs to create trust which is based on collective needs in conserving a resource. In the case of sea turtles, acknowledgement of roles which fishermen play and the unwritten rules which govern their resource use, can create a better policy for sea turtle governance if the informal rules are integrated into formal rules. This can be facilitated by third parties who bridge gaps and transmit the scientific and traditional knowledge to policy makers who use the knowledge as a foundation for creating a more holistic policy; which incorporates the needs of the community, the environment and creates a stronger step to sea turtle conservation.

## Supplementary Information

Below is the link to the electronic supplementary material.

Supplementary file1 (PDF 462 kb)

## Footnotes

1 https://www.iucnredlist.org.

2 https://www.sprep.org/ioe/regional-marine-species-programme.

3 Management unit: a genetic and demographic independent nesting population managed as a distinct unit (Moritz 1994).

4 Epibiont: an organism that lives on the surface of another organism and by definition is harmless to its host (Lescinsky 2001).

5 Chiefs (**Turaga ni Vanua**) are hereditary leaders in iTaukei villages chosen from the chiefly landowning units (**mataqali**) by the chief-makers (**sauturaga**). Chiefs were responsible for day-to-day village governance and were responsible for the final decision-making during communal meetings. They also regulate the use of resources within customary iTaukei boundaries. (See Appendix S2 in the Supplementary Materials).

6 A **tabua** is the most valuable cultural item in the **iTaukei** society, referred to as **kamunaga** (‘wealth’). **Tabua** was a rare commodity in Fiji prior to the late 1700s, when the Tongans brought whale’s teeth and exchanged them for knowledge and skills on how to construct traditional war canoes (Gatty 2009).

7 The head of tribute refers to the most valued resource presented with customary gifts during **iTaukei** ceremonies performed for weddings, funerals, or other village setting customary gathering.

8 Sea turtles are considered a totem fish because of their underwater marine life. In some parts of Fiji, they are referred to as ‘**ika bula**’ (‘live fish’) because they are marine animals (‘fish’) which remain alive when out of water.

9 **Vula I katakata** refers to the iTaukei calendar period which includes the summer months of November through to March. This period is signified by the ripening of native fruit trees, spawning of land crabs (*Cardisoma carniflex*), sea turtle nesting period and aggregation of baitfish which attract large finfish. This is also the period in which iTaukei fishers carry out group fishing (**yavirau**).

10 Ethno-species: vernacular names used in folk classification that can match, under-differentiate or over-differentiate as compared to scientific species (Berlin 1992).

11 1997–2000 (3-year moratorium), 2002–2007 (5-year moratorium), and 2009–2018 (10-year moratorium).

12 Information on how respondents are recruited to SurveyMonkey is available here: www.surveymonkey.com/mp/audience.

13 Root of the kava plant (*Piper methysticum*) used as a ceremonial or social beverage in Fiji.

14 https://lmmanetwork.org.

15 **Dua na bi** is equivalent to ten sea turtles in the **iTaukei** traditional counting system. **Bi** refers to the standing cages in which sea turtles are kept after being captured. Ten adult sea turtles can fit in one **Bi**.

16 See Appendix S2 and Appendix S3 in the Supplementary Materials.
