# Supplementary material for: Conservation status and cultural values of sea turtles leading to (un)written parallel management systems in Fiji
Source: Ambio. 2022 Aug 6;51(12):2431–44. doi: 10.1007/s13280-022-01766-4 (PMC9584005; doi:10.1007/s13280-022-01766-4)
Supplement: Supplementary file 1 — Supplementary file1 (PDF 462 kb) [file 13280_2022_1766_MOESM1_ESM.pdf]

Ambio

Supplementary Information
This supplementary information has not been peer reviewed

Title**: Conservation status and cultural values of sea turtles leading to (un)written parallel systems in Fiji**

Table of Contents

Supplementary Map 1: Map of iTaukei villages where sea turtles are a totem 3

Supplementary Table 1: Turtle hunter perceptions …………...............................4 - 6

Supplementary Table 2: Turtle fishing methods Qoma and Denimanu.............. 7 – 8

Supplementary Figure 1: Classical iTaukei society social structure………..………9

Appendix 1: Questionnaire: ……………………………………........……….10 – 13

Appendix 2: The iTaukei social structure and leadership system...……………14 – 15

Appendix 3: Recommendation for sea turtle harvest ...…...……………………..……16

References……………...……………………………………………………………17

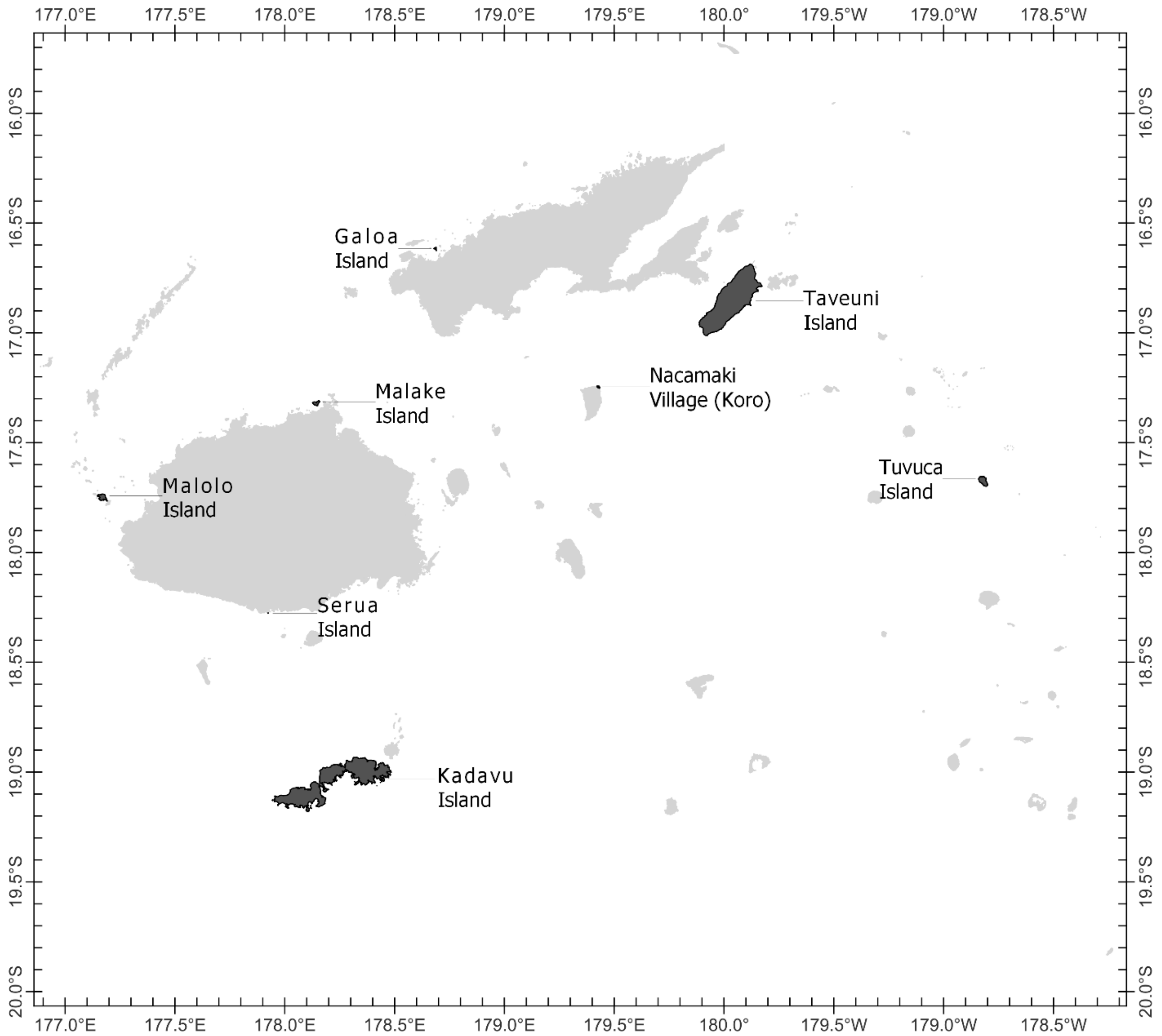

*Supplementary Map 1: Traditional iTaukei villages or islands where sea turtles are considered a totem (**icavuti**) and consumption is forbidden. Source: Pacific Data Hub (https://pacificdata.org/)*

Supplementary Table 1: Past and present fishing methods of Qoma and Denimanu. Descriptions extracted from the collected questionnaires in Denimanu with turtle hunters on February and November 2015 by SSP and SP and the in-depth field interviews conducted by SK and AS in Qoma with older fishers on September 2020 and November 2020.

NB: present fishing methods include the use of spears, torches and fiberglass boats.

Village	Traditional fishing methods	Fishing area	Fishing gear	Time of day	Description of the technique involved in this traditional fishing method
QOMA	**Rabe**<br><br>(*this method is only done when the a chief (Tui Nabulebulewa/Ratu mai Dawasamu or Ratu mai Verata) wants to eat turtle*)	Seagrass beds (**veivutia**)	1. turtle net (**lawasau**)<br>2. one boat<br>3. Sticks (paddles can also be used)	Night	The team of turtle hunters split into two. A team of net holders create a single file parallel to the shore and quietly wade out in knee deep water while holding the net above the water. The sinkers are hanging around the hunters' knees who carry the net and floaters on their shoulders. The second team on the boat creates a disturbance close to scare the sea turtle toward the net and the leader shouts the command to drop the net. The net holders drop the net and remain stationary behind it then shout a special word to indicate the turtle is caught. The leader paddles out to place the turtle in the boat then the net is lifted and the fishing continues until the turtle hunters capture the quota for the chief.
	**Rai ua**	Seagrass beds (**veivutia**)	1. turtle net<br>2. boat and paddle	Day	Selected sea grass area enclosed with fishing net. Boat paddle used to splash the water and chase the sea turtle into the net
	**Taratara**	Seagrass beds (**veivutia**)	1. turtle net<br>2. boat<br>3. sticks (paddles can also be used)	Night	Prior observations done at low tide in the fishing grounds to check for signs of sea turtle activity, and markers are placed to locate the area. Nets are placed at high tide in deeper water. Fishing is carried out when the moon rises. Fishers disturb the water near the sea turtle sleeping area and the sea turtles fleeing from the commotion get caught in the awaiting nets
	**Vakarorogo**	1. Seagrass beds (**veivutia**)<br><br>2. on the reef edge (**bati lili**)	1. boat<br>2. turtle net	Night	Turtle hunters use their canoe to search for sea turtles around the seagrass beds. this method is done when the water is calm so hunter can hear the distinct gasp of a sea turtle as it comes up for air. When the sea turtle is located, hunters rush to catch it and deploy the nets to capture the sea turtle

	**Cocoka / coka vonu** *(new method used by Qoma turtle hunters introduced in the 1980s)*	seagrass beds (**veivutia**)	1. spear with a wooden handle<br>2. fishing line<br>3. fiberglass boat	Day	Turtle hunters navigate area where sea turtles float around after they eat. When they spot a sea turtle, they drop anchor. The fishing line is tied to the spear then thrown at the sea turtle. Hunters wait to see the fishing line pull then they dive into the water to capture the sea turtle.

<u>Note on Qoma </u>: other fishing methods used in Qoma were described by Jit (2007) and Veitayaki (1990) in their thesis and by Roth (1959) in his unpublished notes on the accounts of turtle hunters from Qoma in 1947. The methods described by the three authors not listed above include: **qoli taku, silimi vonu dina, kara vonu, vola lawa, saumoce, siwa, rebai** and **vakacuru luveniwai**.

Village	Traditional fishing methods	Fishing area	Fishing gear	Time of day	Description of the technique involved in this traditional fishing method
DENIMANU	**Nunu**	seagrass beds (**veivutia**)	1.fiberglass boat	Day	Involves a group of turtle hunters spot a turtle from their boat and dive for them, chasing the turtle until it is captured by flipping it on its back. This method is done at high tide.
	**Lawa** (net)	Seagrass beds (**veivutia**)	1.nylon net<br>2.Boat	Day	A group of turtle hunters go out to the spot where a turtle is located drop their net. Another group chases the sea turtle into the net.
	**Vuru** (coconut leaf torches)	Seagrass beds (**veivutia**)	1.coconut leaves<br>2. spear<br>3.boat	Night	Torches are made from coconut leaves and lit to attract sea turtles. when the sea turtles are spotted, they are speared and turtle hunters dive into the water to flip the sea turtle over and lift it into the boat.
	**Nunu bogi**	Seagrass beds (**veivutia**)	1.boat<br>2.torch	Night	Four to five turtle hunters go out on a boat. One holds the torch and the others line up to dive for the turtle and flip it on its back before lifting it into the boat
	**Vakasucu na vonu** (nesting)	Nesting sites	Hand collection so no gear used	Day OR Night	Turtle hunter waits for the sea turtle to come on land and find a nesting spot, then flips the sea turtle over
	**Cocoka**	Lagoon (**toba**)	1.spear<br>2.boat<br>3.torch	Night	Turtle hunters go out at night and free dive then search for turtles and thrust spears at them. Then they flip the sea turtle over and carry it to the boat
	**Cocoka ni siga**	Lagoon (**toba**)	1.spear<br>2.boat	Day	

	**Dakai** (spear guns)<br><br>(this is a modern day method which used to be used in Denimanu but is now banned)	Lagoon (**toba**)	1.speargun<br>2. boat<br>3. torch	Day<br>OR<br>Night	Turtle hunters travel out to the lagoon and when they spot a sea turtle, dive and spear them with a spear gun.

Supplementary Table 2: Turtle hunter perceptions on the restriction of their traditional fishing methods or sea turtle customary practices imposed by the sea turtle legislations and moratoria

**Sea turtle legislation**	**Legislation wording**	**Restriction on traditional fishing methods or customs**
Fisheries Act 1945– [Cap 158]	An act to make provision for the regulation of fishing	<u>*QOMA and DENIMANU*</u> – traditional fishing gear and methods slowly being redundant and knowledge of these fishing methods are no longer passed to the next generation of turtle fishers.
Fisheries Act 1945 – [Cap 158]<br><br>*(Inserted by Regulations 8th June, 1966) (Amended by 87 of 1979)*	**(2)** No person shall be in possession of, sell, offer or expose for sale or export any turtle shell the length of which is less than 455 mm [eighteen inches].	<u>*QOMA*</u>: custom of selling turtles to pay taxes (colonial days) or to asking customers (present) and sharing the income to all members of the household affected.
Fisheries Act 1945 – [Cap 158] *(Amended by 87 of 1979)*	*Spearing of turtle*<br><br>**9**. No person shall harpoon any turtle unless the harpoon is armed with at least one barb of which the point projects not less than 9.5 mm [3/8 inch] from the surface of the shaft, measured at right angles to the long axis of the shaft.	<u>*QOMA*</u>: spear fishing new method of fishing in Qoma. fishers began using it in the 1980s because the traditional method of using nets was time consuming and not lucrative.<br><br><u>*DENIMANU*</u>: the use of specialized turtle spears continued until 2015.<br><br>**NB**: the traditional term used for sea turtle hunting is "**qoli vonu**" which means using nets to harvest sea turtles. Traditionally, only nets were used to capture sea turtles because it is a fishing method used to capture food for paramount chiefs (**qoli vakaturaga**). In the 1800s new fishing methods including spearing of sea turtles and turtle nets was introduced to Fiji by the Tongans.
	Mesh of hand nets<br><br>**13.** The mesh of a hand net may be of any size.	No change
	Mesh of cast nets<br><br>**14.** The mesh of cast nets shall not be less than 30 mm [1.25 inches], wet and stretched.	No change
Fisheries Act 1945 – [Cap 158] *(Amended by Regulations 19th July, 1991)*	*Turtle*<br><br>**20. (1)** No person shall at any time dig up, use, take, sell, offer or expose for sale, or destroy turtle eggs of any species or in any way molest, take, sell, offer or expose for sale, or kill any turtle the shell of which is less than 455 mm [18 inches] in length.	<u>*QOMA*</u>: sea turtle eggs not consumed because no active nesting sites found in Qoma.<br><br><u>*DENIMANU:*</u> fishers no longer hunt for nesting sites where they can collect eggs and capture nesting female turtles.<br><br>**NB**: turtle mating and nesting period between November – February with peak nesting in January and peak hatching in March for hawksbill turtles.<br><br>On the traditional calendar of turtle hunters:

	No person during the months of January, February, November or December in any year shall in any way molest, take, sell, offer or expose for sale, or kill any turtle of any size.	• **Vulaisenidrala** or **Vulaikawakawa** (August) as the turtle mating season.<br>• **Vulaikatakata** (~December – February) as the nesting season.<br>• **Vulaililiwa** (~June – August) is when turtles are hard to find
Fisheries Act 1945– [Cap 158] *(Inserted by Regulations 19th July, 1991)*	**26**. No person shall export from Fiji-<br><br>(b) turtle flesh;<br><br>(c) turtle shell unless worked into jewelry or otherwise processed into a form approved by the Permanent Secretary for Primary Industries and Cooperatives."	<u>*QOMA and DENIMANU:*</u> not affected by this legislation as they harvest and use the sea turtle products locally.
Offshore Fisheries Management Regulation (OFMR) 2014	– does not allow any of the mentioned above unless of course a permit application is made to the Ministry of Fisheries and approved by the Director of Fisheries for traditional use only.	<u>*QOMA and DENIMANU:*</u> custom where a chief presents kava to the **gonedau** still used, however, today anyone wanting to eat sea turtle meat either presents kava with their intention for sea turtle harvest directly to the chief then await approval before they go and harvest the turtle without the **gonedau** or call their relatives who can capture sea turtles and bring it to them.<br><br>**NB: Gonedau** – traditional fisher clans in an iTaukei village setting. They are led by the **Tunidau** (title of the head of fisher clans). A paramount chief has several iTaukei villages who act as the traditional fisher clans and their duty is to provide marine and aquatic resources for chiefly gatherings when they are informed of the gathering or when they are requested by their paramount chief/village chief that he/she wants to eat a certain delicacy like a sea turtle.

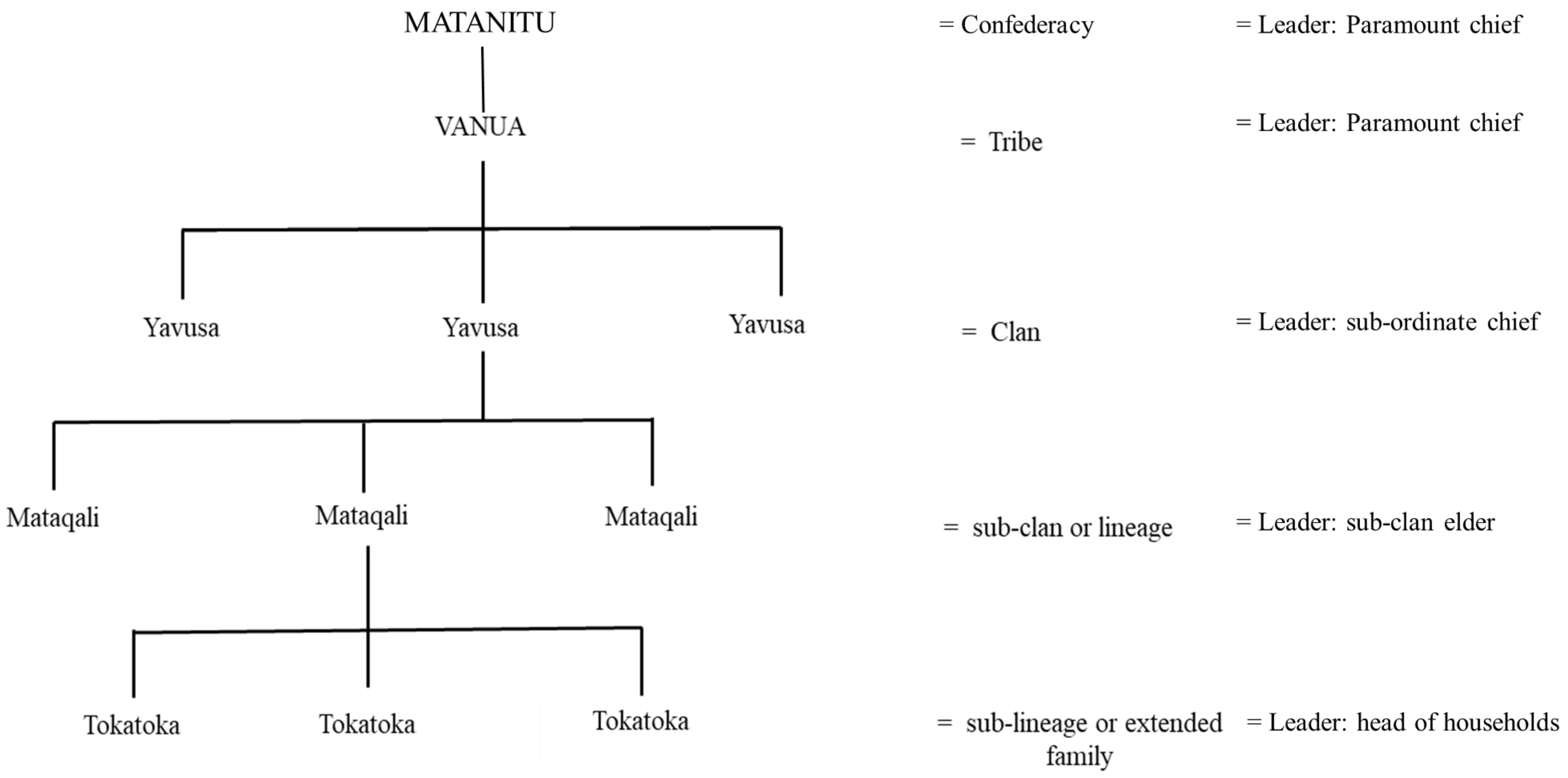

***Supplementary Figure 1**: Classical iTaukei society social structure. Adopted from Ravuvu (1983)*

Appendix 1: Quetionnaire

CONFIDENTIALITY AGREEMENT

I agree to keep personal data concerning this project safe and secure. If used for a manuscript to be submitted to a journal, data will be grouped, and no personal recognition stated. Information about this project will be retained by Dr. Susanna Piovano, USP. The support from the community will be acknowledged in every manuscript coming out from this research.

Full name of interviewer:	
Signature:	
Date:	

INDIVIDUAL INTERVIEW ON

ASSESSMENT OF CAPTURE AND COSUMPTION OF SEA TURTLES

Island:		Village:	
Interviewed	Full name		
	Gender		
	Age		
	Occupation		

QUESTIONS

1. How do you call the turtle's species in your dialect?

Hawksbill	
Green	
Loggerhead	

2. How often sea turtles were/are caught and where? (Use both table and map. For each single map, write the number of turtles caught by unit of time, and write which kind of environment it is – e.g: seagrass prairie, coral reefs, mangroves, etc.)

		**Weekly**			**Monthly**			**Yearly**		
		**30 yrs ago**	**15 yrs ago**	**Now**	**30 yrs ago**	**15 yrs ago**	**Now**	**30 yrs ago**	**15 yrs ago**	**Now**
**Hawksbill**	Eggs									
	Juvenile									
	Adults									
**Green**	Eggs									
	Juvenile									
	Adults									

**Loggerhead**	Eggs									
	Juveniles									
	Adults									

3. What was/is the main purpose of these captures? (use both table and map. On the map, write the information using different color)

Purpose of turtles' capture	Species target			No. of turtles captured			Turtles' life stage			Locality of harvest (use numbers on map)		
				H	G	L	H	G	L	H	G	L
Food for the family	H	G	L									
As an exchange for other kind of food	H	G	L									
As an echange for other commodities	H	G	L									
To sell it within the village	H	G	L									
To sell it at the market	H	G	L									
To sell it to middle men	H	G	L									
Upon chief request	H	G	L									
Wedding	H	G	L									
Birthdays	H	G	L									
Christmas	H	G	L									
New Year	H	G	L									
Easter	H	G	L									
Funeral	H	G	L									
Cementing	H	G	L									
Church funding event	H	G	L									
Other:	H	G	L									
Other:	H	G	L									

4. How was/is decided whether to target eggs, juvenile or adult turtles?

**30 years ago**	
**15 years ago**	
**Now**	

5. How was/is hunting organized?

		30 years ago	15 years ago	Now
**Nests**	Were spotted by change and eggs harvested			
	Were actively search for and eggs harvested			
**Turtles**	Were spotted by change and captured			
	Were actively searched and captured			
**Capture of turtles**	Was done by the only person indicated by the chief			
	Was done by single fishermen			
	Was done by group of fishermen			
	Involves the whole village, including women			

6. How were/are turtles captured?

	30 years ago	15 years ago	Now
By hand at the beach (while they nested)			
By spear diving at night			
By spear diving at day			
By free diving at night			
By free diving at day			
By rodeo			
By fixed nets			
By active nets			

7. how many people in your community captured/capture sea turtles?

	30 years ago	15 years ago	Now
0 – 25% (just a few people)			
25 – 50% (less than half the village)			
50 – 75% (more than half the village			
75 – 100% (almost everybody)			

8. How many people in your community eaten/eat sea turtles?

	30 years ago	15 years ago	Now
0 – 25% (just a few people)			
25 – 50% (less than half the village)			

50 – 75% (more than half the village			
75 – 100% (almost everybody)			

9. what is your perception about sea turtles with respect to 15 and 30 years ago?

	**Compared to 30 years ago**			**Compared to 15 years ago**		
	**Increased**	**Decreased**	**Same**	**Increased**	**Decreased**	**Same**
Number of nests						
Number of juvenile turtles						
Number of adult turtles						

10. What do you think is the reason for that? Do you have any suggestion?

____

____

____

____

____

11. What do you think it can be done to improve the situation?

____

____

____

____

____

12. The current Moratorium will end in three years. What do you think will happen once sea turtles will no longer be protected? What is your suggestion?

____

____

____

____

____

Vinaka vakalevu

Appendix 2:

The iTaukei social structure and leadership system

Fiji's polity has undergone complex changes over time. Kinship groups including the **matanitu** (confederacy), **vanua** (tribe), **yavusa** (clan), **mataqali** (sub-clan) and **tokatoka** (lineage/extended family) (Figure S1) are interdependent and bound together through links created by their ancestors, places and politics (Black 2010).

Extended family groups (**tokatoka**) make up the foundation of a village (**koro**). Several families together make up a **mataqali** which is formed through matrilineal and patrilineal kinship. Within one **koro**, there can be several **mataqali** of different descent. Over time, kinship groups form and reform through migration and resettlement of different **mataqali**, before they merge to create a **yavusa**. Capell and Lester (1941) noted that a **yavusa** had the same **icavuti** (totems) which was used as an identity. Each **yavusa** has its own chief (**turaga ni yavusa**).

Over time, a **yavusa** formed bonds and strengthened ties with one another creating the **vanua**, which is headed by a paramount chief (**turaga/marama bale**). A paramount chief is the highest ranking chief, to whom sub-chiefs pay tribute. At the apex of the **vanua** is the **matanitu** (confederacy) also led by a paramount chief. At the time of contact period with the missionaries in Fiji, the **matanitu** were very powerful. According to Thomas (1986), the **matanitu** consisted a "regionally extensive system of relations of subordination, allegiances whose chiefly place interacted continuously with groups within the domain in terms of these relationships as well as with other **matanitu** and interdependent groups" (Thomas 1986: 64). During early contact period with missionaries, seven **matanitu** dominated iTaukei society headed by paramount chiefs, who are still present today.

These seven include Bau, Rewa, Cakaudrove, Bua, Macuata, Verata and Lau. The paramount chiefs of these seven **matanitu** include:

1. Kubuna na Tui Kaba, na Vunivalu (Bau)
2. Burebasaga na Gone Marama Bale na Roko Tui Dreketi, na Vunivalu (Rewa)
3. Lalagavesi na Turaga na Tui Cakau, nai Sokula (Cakaudrove)
4. Cakaunitabua na Turaga na Tui Bua (Bua)
5. Caumatalevu na Turaga na Tui Macuata (Macuata)
6. Naisanokonoko na Turaga na Ratu (Verata)
7. Vuanirewa na Turaga na Tui Nayau (Lau)

Appendix 3: Recommendation for sea turtle harvest

Brewster (1922) in his accounts describes the turtle fleets in Fiji, which were made up of **gonedau** (traditional fishermen)/turtle hunters led by the Tunidau (head fisherman) sent out to capture sea turtles. The ordinary levy which was expected by the paramount chief was 10 sea turtles for each canoe in the fleet. Once all canoes procured 9 sea turtles, the Tunidau would set a date when the fishing would close. Any canoe short of its expected 10 sea turtles had to have one of its crew killed to make up for the shortage. The 10 sea turtles expected from the paramount chief is termed "**dua na bi**" in the iTaukei traditional counting system.

Our recommendation, is that instead of using the turtle fleets, the paramount chiefs are allocated **dua na bi** and the burden of capturing 10 sea turtles is shared among the paramount chief's **gonedau**. The sea turtles are then kept in sea pens or tidal pools until they are needed.

References

Black, H. 2010. Sere dina ni Lotu Wesele e Viti: “True Songs”. The history, culture and music of Fijian Methodist Indigenous Liturgy. Doctor of Philosophy, Canberra: Australian National University. doi:10.25911/5d77878f9eac3.

Brewster, A. B. 1922. *The hill tribes of Fiji. A record of forty years intimate connection with the tribes of the mountainous interior of Fiji with a description of their habitats in war and peace, methods of living, characteristics, mental and physical, from the days of cannibalism to the present time*. London: Seeley, Service & Co. Ltd.

Capell, A., and R. H. Lester. 1941. Local divisions and movement in Fiji. *Oceania* 12: 21–48. doi:10.1002/j.1834-4461.1941.tb00344.x.

Jit, J. 2007. Status of sea turtles conservation in Fiji: assessment of the international, regional and national focus. Masters Thesis, Suva: University of the South Pacific.

Ravuvu, S. 1983. *Vaka iTaukei - the Fijian way of life*. Suva: Suva Printing.

Roth, G. K. 1959. Fishing (2). National Library of Australia. Mfm M 2780-2793-Papers of George Kingsley Roth (as filmed by the AJCP) [microform] : [M2780-2793], 1874-1959./Series ADD 8780/File 80. Box 8/Fishing (2). Australian Joint Copying Project onlnie portal.

Thomas, N. P. G. 1986. *Planets around the sun: dynamics and contradictions of the Fijian Matanitu*. Oceania Monographs 31. Sydney, Australia: University of Sydney.

Veitayaki, J. 1990. Village level fishing - a case study of Qoma. Masters Thesis, Suva: University of the South Pacific.
